# PPP1CB-Related Noonan Syndrome with Loose Anagen Hair: A Systematic Review

**DOI:** 10.3390/genes17060603

**Published:** 2026-05-26

**Authors:** Giuseppe Reynolds, Marta Calvo, Maria Luca, Stefania Massuras, Federico Rondot, Simona Cardaropoli, Alessandro Mussa

**Affiliations:** 1 Department of Public Health and Pediatrics, University of Turin, 10126 Turin, Italy; giuseppe.reynolds@unito.it (G.R.); marta.calvo@unito.it (M.C.); federico.rondot@unito.it (F.R.); simona.cardaropoli@unito.it (S.C.); 2 Postgraduate School of Pediatrics, Department of Public Health and Pediatrics, University of Turin, 10126 Turin, Italy; 3 Ph.D. Program in Biomedical Sciences and Oncology, Department of Public Health and Pediatrics, University of Turin, 10126 Turin, Italy; 4 Pediatric Clinical Genetics, Regina Margherita Children’s Hospital, 10126 Turin, Italy; maria.luca@unito.it (M.L.); stefania.massuras@unito.it (S.M.)

**Keywords:** *PPP1CB*, NSLH2, Noonan syndrome-like disorder with loose anagen hair, RASopathy, SHOC2-MRAS-PP1C complex

## Abstract

**Background**: *PPP1CB*-related Noonan syndrome-like disorder with loose anagen hair type 2 (NSLH2; OMIM #617506) is a rare RASopathy caused by pathogenic variants in PPP1CB, encoding the catalytic beta subunit of protein phosphatase 1 (PP1C). Since its first description in 2016, only a limited number of patients have been reported, leaving the full phenotypic spectrum and genotype–phenotype correlations largely undefined. **Objectives**: To systematically review the clinical, molecular, and functional characteristics of NSLH2, we define its phenotypic spectrum, explore genotype–phenotype correlations, and summarize current evidence on therapeutic management. **Methods**: A systematic literature search was conducted across PubMed/MEDLINE, Embase, Web of Science, and Google Scholar, supplemented by searches of Orphanet, OMIM, and ClinVar, from 2016 to 2026. Studies reporting patients with pathogenic or likely pathogenic variants in PPP1CB were included. Individual patient-level data were extracted and analyzed descriptively. Additionally, we report a novel patient identified at our institution. **Results**: Thirty patients from 14 publications were included, harboring nine distinct *PPP1CB* variants. The most frequently identified variant was p.Pro49Arg (n = 17, 56.7%), followed by p.Met182Lys (n = 4, 13.3%) and p.Glu183Ala (n = 3, 10.0%). The majority of variants arose de novo (n = 26, 86.7%). Ectodermal anomalies, predominantly slow-growing and structurally abnormal hair consistent with loose anagen hair, were present in 79.3% of patients. Congenital heart defects were identified in 75.9%, with pulmonary stenosis and atrial septal defect representing the most common lesions. Short stature was documented in 69.2% of cases, and neurodevelopmental delay—encompassing motor and language delay—affected the majority of patients (72.4–84.6%). Brain structural anomalies were detected in 35.7%. Facial dysmorphic features were universal. Macrocephaly was present in 58.6% of cases, intellectual disability was reported in 26.9%, and epilepsy in 6.7%. Three familial cases with inherited p.Met182Lys transmission from an affected mother to three children are described, representing the largest reported familial cluster. **Conclusions**: NSLH2 is a clinically recognizable RASopathy with a consistent core phenotype comprising loose anagen hair, congenital heart defects, short stature, macrocephaly, and neurodevelopmental delay. The p.Pro49Arg variant accounts for the majority of reported cases and appears associated with a broad phenotypic expression. Larger cohorts and functional studies are needed to fully delineate genotype–phenotype correlations and guide therapeutic strategies.

## 1. Introduction

The RASopathies are a clinically and genetically heterogeneous group of neurodevelopmental syndromes caused by germline pathogenic variants in genes encoding components or regulators of the RAS/mitogen-activated protein kinase (MAPK) signaling pathway [1]. This pathway plays a central role in the transduction of extracellular signals governing cell proliferation, differentiation, survival, and migration, and its dysregulation underlies a spectrum of overlapping phenotypes characterized by distinctive craniofacial dysmorphisms, congenital heart defects, short stature, variable intellectual disability, and increased cancer predisposition [2]. The RASopathy group encompasses Noonan syndrome (NS), the most prevalent condition in this spectrum, along with Noonan syndrome with multiple lentigines, cardiofaciocutaneous syndrome, Costello syndrome, Legius syndrome, and Noonan syndrome-like disorder with loose anagen hair (NSLH), among others [3,4].

Noonan syndrome-like disorder with loose anagen hair (NSLH) is a clinically distinct RASopathy subtype characterized by the combination of Noonan syndrome features—including short stature, congenital heart defects, and distinctive facial dysmorphisms—with a pathognomonic ectodermal hallmark: loose anagen hair (LAH), defined as hair that can be extracted painlessly and easily from the scalp, with characteristic ultrastructural abnormalities of the hair shaft and follicle on light and electron microscopy [5]. Two genetically distinct forms are currently recognized. NSLH type 1 (NSLH1; OMIM #607721) is caused by pathogenic variants in SHOC2, encoding the scaffold protein SHOC2, and was the first described form [5,6,7,8]. NSLH type 2 (NSLH2; OMIM #617506) is caused by pathogenic variants in *PPP1CB* and represents a more recently delineated entity.

*PPP1CB* (OMIM *600590) encodes the catalytic beta isoform of protein phosphatase 1 (PP1Cβ), a serine/threonine phosphatase with broad cellular functions including cell cycle regulation, glycogen metabolism, and RNA splicing [9]. In the context of RAS-MAPK signaling, PP1Cβ assumes a critical and specific role as the catalytic subunit of the trimeric SHOC2-MRAS-PP1C holophosphatase complex. This complex, whose structure was resolved at atomic resolution in 2022 by three independent groups [9,10,11], functions as a RAF-activating phosphatase: it dephosphorylates the inhibitory Serine 259 residue of CRAF (RAF1), thereby relieving its autoinhibition and promoting downstream MAPK cascade activation. The complex assembles in a highly specific manner, requiring the simultaneous presence of GTP-loaded MRAS and the scaffold protein SHOC2; disruption of any of these components abrogates complex formation and function (11). Pathogenic variants in all three components of the complex—SHOC2, MRAS, and PPP1CB—have been associated with NSLH or NSLH-like phenotypes, underscoring the central pathogenic role of this complex in this RASopathy subgroup [5,8,11].

NSLH2 was first described in 2016 by Gripp et al., who reported four patients with de novo missense variants in *PPP1CB* presenting with a phenotype closely resembling NSLH1 [8]. Since then, additional patients have been reported in scattered case reports and small case series, progressively expanding the clinical and molecular landscape of this condition. However, the rarity of NSLH2 has precluded systematic characterization of its full phenotypic spectrum, and several critical questions remain unanswered: the complete range and frequency of clinical manifestations, the existence of meaningful genotype–phenotype correlations, the pathogenic mechanisms linking specific *PPP1CB* variants to downstream RAS-MAPK dysregulation, and the optimal therapeutic management—including the role of growth hormone therapy.

The present systematic review aims to: (i) define the clinical phenotypic spectrum of NSLH2 by analyzing all published patients at the individual level; (ii) characterize the molecular landscape of pathogenic *PPP1CB* variants; (iii) explore genotype–phenotype correlations; (iv) synthesize available evidence on functional and structural studies; and (v) summarize current evidence on therapeutic approaches and clinical management. Additionally, we report a novel patient with a previously unreported *PPP1CB* variant (p.Glu183Ala) identified at our institution, further expanding the molecular spectrum of this condition.

## 2. Materials and Methods

**Figure 1:**
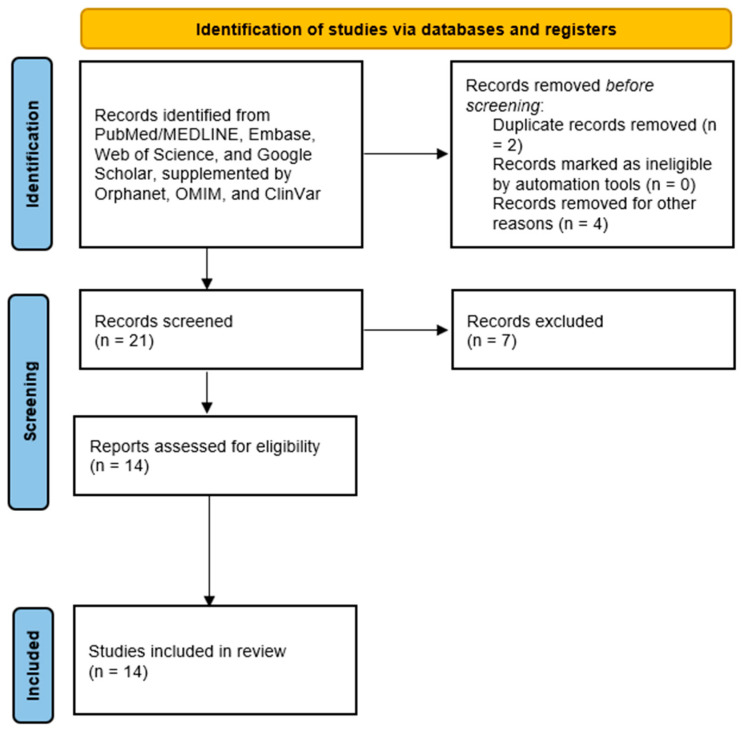
PRISMA 2020 flowchart of the study selection process: the results of the PRISM-based process for the literature selection illustrate the screening process and article counts resulting from our search [12]. Selection and exclusion criteria are more widely reported in the text of the article.

A comprehensive literature search was performed across PubMed/MEDLINE, Embase, Web of Science, and Google Scholar, supplemented by Orphanet, OMIM, and ClinVar, covering the period from January 2016—the year of the first description of NSLH2—through 2026. The search strategy combined the terms “*PPP1CB*”, “Noonan”, “RASopathy”, “loose anagen hair”, “NSLH2”, and “SHOC2-MRAS-PP1C complex”, with no language restrictions. Reference lists of included articles were hand-searched for additional records. This systematic review was conducted and reported according to the PRISMA 2020 guidelines. Following PRISMA guidelines [12], we visually represented the search and selection process in Figure 1. The review was not prospectively registered, and no separate review protocol was prepared.

Studies reporting patients of any age carrying pathogenic or likely pathogenic germline variants in *PPP1CB* were included. Variants of uncertain significance without functional evidence, duplicate patient reports, and somatic variants were excluded. Where the same patient was reported in multiple publications, the most complete source was retained and data supplemented from earlier reports.

Data were extracted at the individual patient level, as recommended for systematic reviews of ultra-rare disorders [13]. Extracted variables encompassed demographic, genetic, perinatal, clinical, therapeutic, and functional data. Additionally, we report a novel patient identified at our institution (Patient 1). Data extraction was performed by one reviewer and verified independently by a second. For our own case (Patient 1), data were collected prospectively as part of routine clinical care. Data synthesis is descriptive; given the small sample size and the predominance of case-level evidence, no formal meta-analysis was performed. Data synthesis is primarily narrative and descriptive. Frequencies and percentages were calculated for categorical clinical variables, and descriptive statistics (median, range) for continuous variables, considering only patients with available data for each parameter. Genotype–phenotype correlations were explored descriptively by stratifying clinical features according to the specific *PPP1CB* variant; given the small sample sizes, formal statistical testing was not performed.

## 3. Results

### 3.1. Our Case

We report a 7-year-old Caucasian boy, the third child of healthy, non-consanguineous parents with two older healthy siblings. Family history was unremarkable. Pregnancy was uneventful; he was born at 39 + 4 weeks of gestation by spontaneous vaginal delivery, with a birth weight of 4270 g (+2.16 SDS). The neonatal period was complicated by mild hypotonia and feeding difficulties, with poor oral intake persisting into early childhood and requiring blended food until school age.

**Figure 2:**
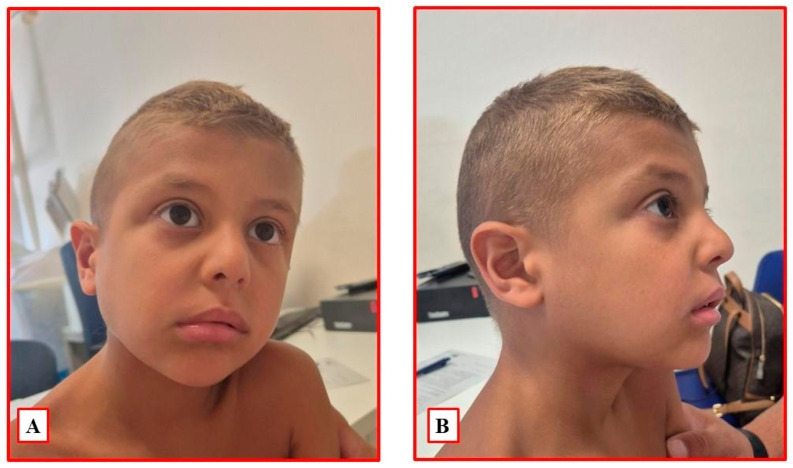
(**A**) Patient 1: front broad nasal tip, mildly down-slanting palpebral fissures. (**B**) Patient 1: low-set ears, coarse and slow-growing hair (photos obtained in our center with written permission of the parents).

At first evaluation at our center at 6 years, dysmorphic features were noted, including a broad nasal tip, slightly low-set ears, and mildly coarse and slow-growing hair. Figure 2 shows facial features of the patient. At the most recent evaluation (age 7 years 9 months), height was 121 cm (9th centile), weight 21 kg (5th centile), and head circumference 51 cm (17th centile). Echocardiography revealed a bicuspid aortic valve, without hemodynamic compromise at the time of diagnosis.

Neurodevelopmental assessment identified delayed motor milestones, with independent walking achieved at 20 months with persistent motor clumsiness, and severe language delay, characterized by the emergence of a few words around 12 months followed by regression and near-absent expressive language at the time of last evaluation, requiring intensive speech therapy. Global neurodevelopmental delay was present; intellectual disability was suspected though formal psychometric testing was not yet conclusive at the time of reporting given the patient’s age and degree of language impairment.

At 26 months of age, the patient developed generalized seizures, for which valproate was initiated with satisfactory seizure control. Brain MRI was not performed at the time of writing. Ophthalmological evaluation was unremarkable. Cryptorchidism of the left testis was surgically corrected.

Whole exome sequencing performed as a singleton identified a heterozygous de novo variant in *PPP1CB*: c.548A>C, p.Glu183Ala, absent from population databases (gnomAD). The variant was classified as Likely Pathogenic according to ACMG/AMP criteria, with the following evidence: PM1 (located within the PP1Cβ catalytic domain, a well-established mutational hotspot for NSLH2-associated variants), PM2 (absent from gnomAD population databases), PM5 (novel missense change at a codon where a distinct pathogenic missense variant has been previously reported: p.Glu183Val), and PP3 (concordant pathogenicity predictions across multiple in silico tools).

### 3.2. Literature Search and Study Selection

The systematic literature search yielded 21 records after deduplication. Following title and abstract screening and full-text review, 14 publications reporting a total of 29 previously described patients with pathogenic or likely pathogenic *PPP1CB* variants were included. Together with our own case (Patient 1), the final cohort comprised 30 patients from 14 publications [8,14,15,16,17,18,19,20,21,22,23,24,25,26].

### 3.3. Molecular Findings

#### 3.3.1. Variant Spectrum

**Figure 3:**
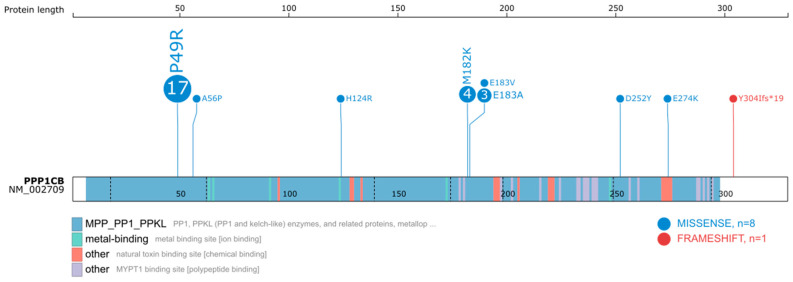
Schematic representation of *PPP1CB* protein domains and distribution of pathogenic variants identified in the present cohort. Lollipop plot depicting the nine disease-associated variants mapped onto the PP1Cβ protein (NM_002709), with bubble size proportional to the number of affected patients. The p.Pro49Arg variant (n = 17) and the cluster of variants at codon 183 (p.Glu183Ala, n = 3; p.Glu183Val, n = 1) represent the two major mutational hotspots. The sole frameshift variant (p.Tyr304Ilefs*19, shown in red) is located in the C-terminal region.

Nine distinct *PPP1CB* variants were identified across the 30 patients (Table 1). All were missense variants, with the exception of one frameshift variant (p.Tyr304Ilefs19, Patient 2) [14]. The most frequently observed variant was p.Pro49Arg (c.146C>G), identified in 17 patients (56.7%), followed by p.Met182Lys (c.545T>A, n = 4, 13.3%), p.Glu183Ala (c.548A>C, n = 3, 10.0%), p.Ala56Pro (c.166G>C, n = 1), p.Glu183Val (c.548A>T, n = 1), p.His124Arg (c.371A>G, n = 1), p.Asp252Tyr (c.754G>T, n = 1), p.Glu274Lys (c.820C>A, n = 1), and p.Tyr304Ilefs19 (c.909dupA, n = 1). Notably, variants at codon 183 (p.Glu183Ala and p.Glu183Val) were collectively observed in 4 patients (13.3%), suggesting this residue as a recurrent mutational hotspot alongside codon 49. Table 1 collects all the reported variants; Figure 3 provides a graphical representation of the amino acid changes in the protein.

#### 3.3.2. Inheritance and Genetic Testing

The vast majority of variants arose de novo (n = 26, 86.7%). Inherited transmission was documented in three cases (Patients 20, 21, and 22), all carrying the p.Met182Lys variant and representing three siblings whose mother (Patient 23) is herself affected—constituting the largest familial cluster reported to date for NSLH2 [21]. Whole exome sequencing (WES), predominantly performed as trio analysis (n = 27, 90.0%), was the diagnostic modality employed in all cases, reflecting the non-specific clinical presentation of NSLH2 and the current centrality of genomic approaches in the diagnostic workup of RASopathies.

### 3.4. Clinical Characterization

The cohort comprised 17 males (56.7%) and 13 females (43.3%). Age at last evaluation ranged from 15 months to 36 years (median 7.0 years, mean 9.1 years). Ethnicity was reported in 15 patients: 7 were of Asian descent (46.7%), 5 Caucasian (33.3%), 2 Hispanic/Latino (13.3%), and 1 of mixed ethnicity (6.7%), reflecting publication patterns and the disproportionate contribution of East Asian case reports rather than population-level prevalence. Clinical features are summarized in Table 2. Detailed characterization of each patient can be found in Supplementary Materials.

#### 3.4.1. Perinatal Findings

Pregnancy was reported as uneventful in 12 of the 17 patients with available data (70.6%), while pathological findings—including increased nuchal translucency, fetal edema, placenta previa, and threatened miscarriage—were documented in 5 (29.4%). Delivery was vaginal in six cases and by caesarean section in five; gestational age at birth was at term in the majority of cases with available data. Neonatal hypotonia was present in 9 patients (30.0%) and feeding difficulties in 10 (33.3%).

#### 3.4.2. Growth

Short stature (height below -2SDS) was documented in 16 of 26 patients with available data (61.5%). Growth hormone (rGH) therapy was initiated in three patients (10.7%), all carrying the p.Pro49Arg variant; response was reported as favorable in two of these, while no efficacy data were available for the third.

#### 3.4.3. Ectodermal and Cutaneous Anomalies

Ectodermal anomalies were the most consistently reported feature after dysmorphic findings, present in 23 of 29 patients with available data (79.3%). Hair abnormalities were the predominant finding, described as slow-growing in the majority of cases, with variable additional features including coarseness, brittleness, hypopigmentation, sparseness, and easy painless extraction—the latter being the hallmark feature of loose anagen hair. Nail anomalies (slow-growing or brittle nails) were reported in a minority of patients. Cutaneous anomalies were present in 13 patients (46.4%) and encompassed a broad spectrum including hyperpigmented lesions (café-au-lait spots, freckling, lentigines), hypopigmented patches, dry or eczema-prone skin, doughy or translucent skin texture, and reduced sweating.

#### 3.4.4. Craniofacial Dysmorphic Features

Dysmorphic facial features were reported universally (30/30, 100%). The most commonly described findings included hypertelorism, low-set and posteriorly rotated ears, broad or depressed nasal bridge with broad nasal tip, and a wide mouth with full lips—a pattern consistent with, but not identical to, that observed in classic Noonan syndrome. Macrocephaly was present in 17 of 29 patients with available data (58.6%). Pectus excavatum or carinatum was documented in a minority of cases.

#### 3.4.5. Congenital Heart Defects

Congenital heart defects (CHDs) were identified in 22 of 29 patients with available data (75.9%), representing one of the most frequent and clinically significant features of NSLH2. The spectrum of cardiac anomalies was broad. Pulmonary stenosis was the most common CHD, consistent with the predominant cardiac finding across the RASopathy spectrum. Atrial septal defect (ASD), predominantly of the ostium secundum type, was frequently co-occurring. Additional malformations included aortic coarctation (Patients 12 and 13), bicuspid aortic valve (Patient 1), dilated aortic root (Patient 7), mitral valve anomalies (Patients 3, 9, 26), ventricular septal defect (Patients 16, 27), patent ductus arteriosus (Patient 28), patent foramen ovale (Patients 10, 13, 15), and, in one patient, a complex malformation including hypoplastic left aortic arch and septo-optic dysplasia (Patient 14). Notably, one patient (Patient 13, p.Asp252Tyr) presented with particularly severe and complex cardiac involvement, including peripheral pulmonary stenosis, aortic coarctation, dilation and tortuosity, and patent foramen ovale.

#### 3.4.6. Neurodevelopmental Profile

Neurodevelopmental involvement was prominent across the cohort. Motor delay was present in 22 of 27 patients with available data (81.5%), with age at independent walking ranging from 20 months to 6 years in those for whom this milestone was reported. Language delay was documented in 22 of 26 patients (84.6%), ranging from mild delay with good recovery following speech therapy to complete absence of verbal communication. Global neurodevelopmental delay was reported in 21 of 29 patients (72.4%). Formal intellectual disability was documented in 7 of 26 patients (26.9%), generally in the mild range, with the exception of Patient 2 (p.Tyr304Ilefs*19), who was non-verbal and had severe developmental impairment. Autism spectrum disorder was reported in only one patient (3.6%). These figures, however, likely underestimate the true prevalence, as the young age of many patients at the time of reporting precluded formal psychometric and neurodevelopmental assessment. Epilepsy was present in two patients (6.7%), both carrying the p.Glu183Ala variant: Patient 1 presented with generalized seizures with onset at 26 months, controlled with valproate, and Patient 17 developed infantile spasms refractory to standard pharmacotherapy, requiring the ketogenic diet.

#### 3.4.7. Neuroimaging

Brain MRI was performed in a subset of patients, with structural anomalies detected in 10 of 28 patients (35.7%). Findings were heterogeneous and included Chiari type 1 malformation (Patient 3), Dandy–Walker malformation with mild lateral and third ventricle enlargement (Patient 6), mild ventriculomegaly (Patients 5, 25, 26), inferior vermian hypoplasia with arachnoid cyst (Patient 4), prominent subarachnoid spaces (Patient 8), septo-optic dysplasia (Patient 14), gliosis or possible migrational abnormality (Patient 12), mildly widened extracerebral spaces (Patient 27), and a complex malformation comprising complete corpus callosum agenesis, ventricular dysmorphisms, hippocampal rotation anomalies, and bilateral periventricular nodular heterotopia (Patient 30, p.Pro49Arg)—the most severe neuroimaging finding in the cohort. Whether septo-optic dysplasia in this patient represents a syndrome-related manifestation of NSLH2 or an independent comorbidity cannot be determined from the available data, given the multifactorial etiology of this condition.

#### 3.4.8. Other Clinical Findings

Ophthalmological anomalies were present in nine patients (31.0%), including proptosis, optic nerve hypoplasia, nystagmus, strabismus, and tear duct anomalies. Cryptorchidism was reported in 7 of 16 male patients (43.8%). Hearing loss, predominantly mild and conductive or sensorineural, was identified in three patients (10.3%). Additional findings reported in individual patients included joint hypermobility, craniosynostosis (Patient 15, p.Pro49Arg), renal anomalies (hydronephrosis, renal cysts, vesicoureteral reflux, double renal pelvis), skeletal anomalies (cervical fusion, clinodactyly, brachydactyly, scoliosis), high-arched palate, and hypogonadotropic hypogonadism and azoospermia in one 16-year-old male patient (Patient 29, p.Pro49Arg). No malignancies were observed in the reported cohort.

## 4. Discussion

### 4.1. Overview

This systematic review provides the most comprehensive characterization to date of *PPP1CB*-related NSLH2, aggregating individual-level clinical and molecular data from 30 patients reported across 14 publications, including a novel case from our institution. Despite the inherent limitations of a literature-based analysis of an ultra-rare condition (including publication bias, heterogeneous reporting, and small sample sizes) the present review delineates a recognizable and clinically coherent phenotype, identifies potentially meaningful genotype–phenotype signals, and highlights critical gaps in current knowledge.

### 4.2. NSLH2 as a Clinically Distinct RASopathy

The clinical profile emerging from this review positions NSLH2 as a recognizable entity within the RASopathy spectrum, sharing core features with classic Noonan syndrome (congenital heart defects, short stature, and distinctive craniofacial dysmorphisms) while being distinguished by a highly prevalent ectodermal signature [4,27]. Slow-growing, structurally abnormal hair consistent with loose anagen hair was present in nearly 80% of patients, representing the most diagnostically distinctive feature of the condition and the one most likely to direct clinical suspicion toward this specific diagnosis. The dermatological and trichological examination therefore assumes particular diagnostic relevance in any child presenting with Noonan-like features, and systematic hair pull test and trichoscopic evaluation should be considered part of the diagnostic workup when NSLH2 is suspected [28].

Macrocephaly, present in nearly 60% of patients, represents another distinguishing feature relative to classic NS, in which macrocephaly is less consistently reported. Conversely, the chest wall deformities and lymphatic anomalies frequently encountered in NS appear to be less prominent in NSLH2, though the limited and heterogeneous reporting across studies precludes definitive conclusions on this point [27,29].

### 4.3. Cardiac Involvement

Congenital heart defects were identified in approximately 76% of patients, confirming cardiac involvement as a cardinal feature of NSLH2. The spectrum of lesions was broad and partially overlapping with that of classic NS, with pulmonary stenosis and atrial septal defect representing the most frequent findings [30,31]. However, several patients exhibited lesions less typical of NS, including aortic coarctation, bicuspid aortic valve, and aortic root dilation. The high prevalence of CHDs underscores the importance of systematic echocardiographic evaluation at diagnosis and structured cardiological follow-up throughout childhood, in line with current recommendations for RASopathies [27,31]. Notably, hypertrophic cardiomyopathy (HCM)—one of the most clinically significant cardiac manifestations in classic Noonan syndrome, reported in approximately 20% of NS cases and associated with increased risk of sudden cardiac death—was not observed in any patient in this cohort [4,32]. This finding is consistent with the broader literature on NSLH2 and may reflect a genuinely lower propensity for myocardial hypertrophy in this RASopathy subtype, though the relatively young age of most reported patients and the limited availability of serial echocardiographic data preclude definitive conclusions. Systematic cardiac surveillance throughout childhood and adolescence remains warranted [27].

### 4.4. Neurodevelopmental Profile and Brain Anomalies

Neurodevelopmental involvement emerged as a prominent and clinically significant dimension of NSLH2. Motor and language delay affected the large majority of patients, and while formal intellectual disability was documented in only approximately 27%, generally in the mild range, the overall neurodevelopmental burden appears greater than in classic NS, in which cognitive development is typically mildly affected [33,34]. This observation has direct implications for clinical management, as it argues for early and systematic neurodevelopmental assessment, prompt referral for speech and physiotherapy, and structured school support in all patients with NSLH2.

Brain structural anomalies on MRI were detected in over one-third of patients, with a broad and heterogeneous spectrum of findings. While no single malformation pattern was predominant, the presence of structural anomalies in a substantial proportion of patients—including, in one case, a complex malformation comprising corpus callosum agenesis and periventricular nodular heterotopia—suggests that brain MRI should be considered as part of the initial diagnostic workup in all patients with NSLH2, particularly those presenting with neurodevelopmental delay or epilepsy. This recommendation is not currently included in published management guidelines for RASopathies, which were largely developed before NSLH2 was fully characterized [27,35].

Epilepsy was observed in only two patients in this cohort (6.7%), a frequency lower than might be expected given the high prevalence of brain structural anomalies. Crucially, both affected patients carried the p.Glu183Ala variant, raising the possibility of a variant-specific epilepsy risk within NSLH2—a hypothesis that warrants prospective investigation in larger cohorts.

### 4.5. Growth Hormone Therapy

Growth hormone therapy was initiated in only three patients in this cohort, precluding any systematic evaluation of its efficacy in NSLH2 [8,20,22]. Available data suggest a favorable growth response in two treated patients, while in another one no information is available about efficacy, consistent with the established efficacy of rGH therapy in NS and other RASopathies [20,22]. Prospective data on rGH response and safety in NSLH2 are needed.

### 4.6. Malignancies

No malignant neoplasm has been reported in any patient with NSLH2 to date. While RASopathies as a group carry an increased baseline risk of certain malignancies—most notably juvenile myelomonocytic leukemia in Noonan syndrome—the current evidence does not support a similarly elevated cancer risk in NSLH2 [36,37,38]. However, given the small cohort size, the young age of most reported patients, and the limited duration of follow-up, this observation should be interpreted with caution, and oncological surveillance remains a relevant consideration in long-term clinical management.

### 4.7. Structural and Functional Basis of the SHOC2-MRAS-PP1C Complex: Implications for PPP1CB Pathogenic Variants

The pathogenic mechanism underlying NSLH2 converges on dysregulation of the SHOC2-MRAS-PP1C (SMP) holophosphatase complex and consequent hyperactivation of the RAS-MAPK pathway. In 2022, three independent groups resolved the atomic structure of this trimeric complex, providing a comprehensive framework for understanding how PPP1CB pathogenic variants alter complex function [9,10,11].

The SMP complex assembles in a highly cooperative manner: SHOC2 acts as the central scaffold, engaging both MRAS and PP1Cβ through its concave leucine-rich repeat (LRR) surface, while creating a mutually dependent protein–protein interaction network involving all three subunits simultaneously. PP1Cβ alone cannot form stable binary complexes with either SHOC2 or MRAS; stable ternary complex formation strictly requires GTP-loaded MRAS, which undergoes a conformational shift in its switch I and switch II regions that is essential for both SHOC2 and PP1C engagement. Once assembled, the holophosphatase selectively dephosphorylates the inhibitory Ser259 residue of CRAF (Ser365 in BRAF), relieving RAF autoinhibition and promoting downstream MEK-ERK activation—an activity that is enhanced 10- to 30-fold relative to apo-PP1C.

All *PPP1CB* pathogenic variants identified in NSLH2 patients are missense variants (with the exception of the frameshift p.Tyr304Ilefs*19) and map to structurally and functionally critical positions within the PP1Cβ catalytic subunit. The structural data provide a direct mechanistic rationale for their gain-of-function effect.

The p.Pro49Arg variant, the most frequent in our cohort (n = 17, 56.7%), substitutes a conserved proline at the SHOC2-PP1C binding interface. In the crystal structure of the SMP complex, the equivalent residue in PP1CA (Pro50) is located at the surface of the SHOC2-PP1C interface, adjacent to the RVxF binding pocket. The p.Pro49Arg substitution introduces a positively charged arginine residue that forms a de novo hydrogen bond with SHOC2-N225 and additional ionic interactions with SHOC2-E224, resulting in a marked increase in SHOC2-PP1C binding affinity—measured by surface plasmon resonance as approximately a 4-fold improvement in KD relative to wild-type PP1C. This enhanced interfacial stability translates into greater SMP complex formation, sustained RAF dephosphorylation, and amplified MAPK signaling. The recurrence of this variant across multiple unrelated families and ethnic backgrounds is therefore consistent with its strong structural effect at a critical interaction surface.

The p.Glu183Ala variant (n = 3 in our cohort, including Patient 1) affects a glutamate residue located within the catalytic domain of PP1Cβ at the SHOC2-PP1C LRR interface. In the SMP crystal structure, the equivalent PP1CA residue Glu184 forms an electrostatic repulsion with SHOC2-E155, which in the wild-type complex partially destabilizes the SHOC2-PP1C interaction. The p.Glu183Ala substitution eliminates this charge–charge repulsion, thereby relieving an intrinsic inhibitory constraint on complex assembly and increasing the apparent affinity of SMP complex formation by approximately 4-fold (KD ~35 nM versus ~140 nM for wild-type). Notably, the adjacent residue Met182 (affected by the p.Met182Lys variant, n = 4 in our cohort) also maps to the MRAS-PP1C and SHOC2-PP1C interface region, suggesting that codon 183—along with the adjacent codon 182—represents a structurally critical hotspot where multiple distinct substitutions can each independently enhance complex assembly. The clustering of NSLH2-associated variants at codons 49 and 182–183 thus reflects the structural architecture of the SMP complex, in which these residues occupy positions of disproportionate functional importance at subunit interfaces.

The two additional rare variants for which structural data are available—p.Ala56Pro (n = 1) and p.Asp252Tyr/p.Glu274Lys (n = 1 each)—map to distinct structural loci. The p.Ala56Pro substitution is located within a loop adjacent to the RVxF binding site of PP1C, though no measurable change in SMP complex affinity was observed in vitro, suggesting its pathogenic effect may operate through subtler conformational perturbations. By contrast, p.Asp252Tyr and p.Glu274Lys map to the acidic and hydrophobic channels of the PP1C active site, respectively—grooves that line the substrate-docking interface for the RAF CR2-pS peptide. These variants may therefore alter substrate selectivity or catalytic geometry rather than complex assembly per se, potentially explaining the atypical and severe clinical presentations observed in the corresponding patients (Patients 13 and 14).

Finally, the sole frameshift variant in our cohort (p.Tyr304Ilefs*19, Patient 2) truncates the PP1Cβ C-terminal domain, a region that, while not directly at the SHOC2 or MRAS interface, contributes to the overall structural integrity of the catalytic subunit. The profoundly severe neurodevelopmental phenotype of this patient may reflect a loss-of-function or dominant-negative mechanism mechanistically distinct from the gain-of-phosphatase-function postulated for the missense variants, a hypothesis that awaits functional validation.

### 4.8. Genotype–Phenotype Correlations

The genotype–phenotype analysis, while limited by sample size, generated several observations of potential clinical relevance. The p.Pro49Arg variant, accounting for over half of all reported cases, was associated with a broad and variable phenotypic expression, encompassing the full spectrum of NSLH2 manifestations.

An intriguing genotype–phenotype signal identified in this cohort concerns epilepsy and the p.Glu183Ala variant. Both patients with epilepsy in this series carry this variant, yielding a frequency of two out of three (67%) within this group compared to 0% in p.Pro49Arg and p.Met182Lys carriers. Notably, even excluding Patient 1—contributed by our own institution in the present review—the signal persists, with one of the remaining two p.Glu183Ala carriers affected by epilepsy versus none in the other variant groups.

However, several caveats must be acknowledged. The total number of p.Glu183Ala patients remains extremely small (n = 3), and one of the two epilepsy cases is Patient 1, contributed by our own institution in the present review. Furthermore, the two affected patients display markedly different seizure phenotypes—generalized seizures responsive to valproate versus drug-resistant infantile spasms requiring the ketogenic diet—highlighting substantial within-variant phenotypic heterogeneity. Taken together, the p.Glu183Ala–epilepsy association should be regarded as a preliminary, hypothesis-generating observation requiring prospective validation in larger independent cohorts before any clinical implications can be drawn.

The p.Met182Lys familial cluster (three siblings and their affected mother, all sharing the same variant) provides unique insights into the inheritance dynamics and intrafamilial variability of NSLH2 [21]. The documented vertical transmission confirms that autosomal dominant inheritance with preserved reproductive fitness is possible in this condition, consistent with the relatively mild overall phenotype observed in some carriers. This has practical implications for genetic counseling of affected individuals and their families. Furthermore, the true prevalence of familial NSLH2 is likely considerably higher than currently reported, as mildly affected individuals may go unrecognized in the absence of systematic family evaluation, representing a potential source of significant ascertainment bias in the existing literature. Detailed genotype–phenotype comparisons across the three main variant groups are presented in Table 3.

The sole frameshift variant in the cohort (p.Tyr304Ilefs*19, Patient 2) was associated with the most severe neurodevelopmental phenotype, suggesting that loss-of-function mechanisms may produce a distinct and potentially more severe clinical course compared to the gain-of-phosphatase-function postulated for missense variants [14]. This hypothesis, however, requires functional validation.

### 4.9. Comparison with NSLH1

A direct comparison between NSLH2 and NSLH1—the *SHOC2*-related form—is instructive both clinically and mechanistically. The two conditions share the defining ectodermal hallmark of loose anagen hair and the broader Noonan-like phenotypic background, reflecting their common pathogenic convergence on the SHOC2-MRAS-PP1C complex. However, several phenotypic differences have been noted in the literature. NSLH1 has been reported to show a higher prevalence of cognitive impairment, more frequent skin anomalies including darkly pigmented skin and eczema, and a greater tendency toward distinctive behavioral features [5,7,39]. Whether NSLH2 carries a comparably elevated neurodevelopmental burden, or whether the present cohort overestimates it due to ascertainment bias toward more severely affected patients, cannot be determined from the current evidence. Prospective, unbiased cohort studies directly comparing NSLH1 and NSLH2 are needed.

### 4.10. Limitations

Several limitations of this review must be acknowledged. First, the evidence base consists almost exclusively of case reports and small case series, with the inherent risks of publication bias toward unusual or severe presentations. Second, the completeness of clinical data varied substantially across reports, limiting the precision of frequency estimates for several features. Third, the small overall cohort size—particularly for individual variant subgroups—constrains the power of genotype–phenotype analyses, and the associations reported here should be considered hypothesis-generating rather than conclusive. Fourth, long-term outcome data are largely absent, as the median age at last evaluation was only 7 years; the natural history of NSLH2 in adulthood remains essentially unknown, with Patient 29 representing the only adult patient followed beyond age 20.

Fourth, long-term outcome data are largely absent. Only four adult patients have been reported, and detailed endocrine and reproductive follow-up data are limited. Although hypogonadotropic hypogonadism with azoospermia was described in one 16-year-old male patient, this isolated observation should be interpreted with caution, as causality cannot be inferred from a single case and alternative contributors were not systematically excluded. Conversely, preserved reproductive fitness is documented in one affected adult female, indicating that infertility cannot currently be considered a consistent feature of NSLH2. Systematic pubertal, endocrine, and reproductive follow-up of adolescent and adult patients will be needed to better define this aspect of the natural history.

Two limitations are specific to our index case. Brain MRI has not yet been performed, due to limited access to neuroimaging during the patient’s early years in a low-income country and subsequent non-compliance after our recommendation; this is acknowledged as an inconsistency with our own diagnostic recommendations. Formal hair examination was also not performed, reflecting current gaps in our institutional clinical pathway that this review has prompted us to address.

## 5. Conclusions

*PPP1CB*-related NSLH2 is a clinically recognizable RASopathy defined by a consistent core phenotype comprising slow-growing and structurally abnormal hair consistent with loose anagen hair, congenital heart defects, short stature, macrocephaly, and neurodevelopmental delay. Dysmorphic facial features are universal. The p.Pro49Arg variant accounts for the majority of reported cases, while codon 183 emerges as a second recurrent mutational hotspot. A potential association between the p.Glu183Ala variant and epilepsy, and the absence of macrocephaly in this variant group, represent novel genotype–phenotype signals warranting prospective validation.

From a clinical management perspective, our findings support systematic echocardiographic evaluation, brain MRI, and early neurodevelopmental assessment in all patients at diagnosis. Given the high frequency of ectodermal involvement in NSLH2, systematic hair assessment, including a hair pull test and trichoscopy, may represent a simple and low-cost addition to the clinical evaluation of patients with suspected NSLH2 or related RASopathies. The possible association between p.Glu183Ala and epilepsy is intriguing but preliminary, and requires confirmation in larger cohorts before any specific clinical recommendation can be made. The evidence on growth hormone therapy remains limited, and prospective safety and efficacy data are needed.

The present review underscores the critical importance of international data sharing and collaborative registries for ultra-rare conditions such as NSLH2, in which no single center can accrue sufficient case numbers to generate robust clinical evidence. Functional studies leveraging the recently resolved crystal structure of the SHOC2-MRAS-PP1C complex offer a promising avenue for mechanistic dissection of variant-specific effects and, ultimately, for the rational design of targeted therapeutic strategies.

## Figures and Tables

**Table 1:** *PPP1CB* pathogenic variants identified in patients with NSLH2. All variants classified as pathogenic or likely pathogenic according to ACMG/AMP guidelines. Variant nomenclature according to NM_002709.3 (*PPP1CB*). The p.Met182Lys inherited cases comprise three siblings (Patients 20–22) who inherited the variant from their affected mother (Patient 23).

Protein Variant	cDNA Change	Type	N (%)	Inheritance	Exon
p.Pro49Arg	c.146C>G	Missense	17 (56.7%)	De novo (17/17)	2
p.Met182Lys	c.545T>A	Missense	4 (13.3%)	De novo (1/4); Inherited (3/4)	6
p.Glu183Ala	c.548A>C	Missense	3 (10.0%)	De novo (3/3)	6
p.Glu183Val	c.548A>T	Missense	1 (3.3%)	De novo	6
p.Ala56Pro	c.166G>C	Missense	1 (3.3%)	De novo	2
p.His124Arg	c.371A>G	Missense	1 (3.3%)	De novo	4
p.Asp252Tyr	c.754G>T	Missense	1 (3.3%)	De novo	8
p.Glu274Lys	c.820C>A	Missense	1 (3.3%)	De novo	9
p.Tyr304Ilefs*19	c.909dupA	Frameshift	1 (3.3%)	De novo	10

**Table 2:** Clinical features of 30 patients with *PPP1CB*-related NSLH2.

Feature	n/N	%
Dysmorphic facial features	30/30	100
Short stature	16/26	61.5
Growth hormone therapy	3/28	10.7
Macrocephaly	17/29	58.6
Pectus excavatum/carinatum	8/30	26.7
Pathological pregnancy	5/17	29.4
Neonatal hypotonia	9/30	30.0
Feeding difficulties	10/30	33.3
Ectodermal anomalies (hair/nails)	23/29	79.3
Cutaneous anomalies	13/28	46.4
Congenital heart defects (any)	22/29	75.9
—Pulmonary stenosis	8/29	27.5
—Atrial septal defect	7/29	24.1
—Patent foramen ovale	3/29	10.3
—Aortic coarctation	2/29	6.9
—Other CHD	6/29	20.7
Language delay	22/26	84.6
Motor delay	22/27	81.5
Global neurodevelopmental delay	21/29	72.4
Intellectual disability	7/26	26.9
Autism spectrum disorder	1/28	3.6
Ophthalmological anomalies	9/29	31.0
Brain structural anomalies (MRI)	10/28	35.7
Epilepsy	2/30	6.7
Cryptorchidism (males only)	7/16	43.8
Hearing loss	3/29	10.3

**Table 3:** Genotype–phenotype correlations across the three most frequent *PPP1CB* variants.

Feature	p.Pro49Arg (n = 17)	p.Met182Lys (n = 4)	p.Glu183Ala (n = 3)
Congenital heart defects	15/17 (88.2%)	1/4 (25.0%)	3/3 (100.0%)
Short stature	12/15 (80.0%)	4/4 (100.0%)	2/3 (66.7%)
Macrocephaly	11/17 (64.7%)	3/4 (75.0%)	**0/3 (0.0%)**
Ectodermal anomalies	13/16 (81.3%)	4/4 (100.0%)	2/3 (66.7%)
Neurodevelopmental delay	14/17 (82.4%)	4/4 (100.0%)	1/3 (33.3%)
Brain structural anomalies	8/17 (47.1%)	3/4 (75.0%)	0/3 (0.0%)
Intellectual disability	4/15 (26.7%)	1/4 (25.0%)	1/3 (33.3%)
Epilepsy	0/17 (0.0%)	0/4 (0.0%)	**2/3 (66.7%)**
Ophthalmological anomalies	5/16 (31.3%)	1/4 (25.0%)	1/3 (33.3%)
Cryptorchidism (males)	5/10 (50.0%)	0/1 (0.0%)	1/1 (100.0%)

## Data Availability

The original contributions presented in this study are included in the article/Supplementary Materials. Further inquiries can be directed to the corresponding author.

## Supplementary Materials

The following supporting information can be downloaded at: https://www.mdpi.com/article/10.3390/genes17060603/s1. The Table in the Supplementary Materials summarizes the clinical and genetic data of 30 patients carrying pathogenic variants in the *PPP1CB* gene.

## Abbreviations

The following abbreviations are used in this manuscript:

NSLH2	Noonan syndrome-like disorder with loose anagen hair type 2
PP1C	Protein phosphatase 1
MAPK	Mitogen-activated protein kinase
NS	Noonan syndrome
LAH	Loose anagen hair
NSHL1	Noonan syndrome-like disorder with loose anagen hair type 1
PP1Cβ	Beta isoform of protein phosphatase 1
SDS	Standard deviation score
MRI	Magnetic resonance imaging
ACMG	American college of medical genetics and genomics
AMP	Association for molecular pathology
WES	Whole exome sequencing
rGH	Recombinant growth hormone
CHDs	Congenital heart defects
ASDs	Atrial septal defects
